# BICS01 Mediates Reversible Anti-seizure Effects in Brain Slice Models of Epilepsy

**DOI:** 10.3389/fneur.2021.791608

**Published:** 2022-01-06

**Authors:** Gareth Morris, Mona Heiland, Kai Lamottke, Haifeng Guan, Thomas D. M. Hill, Yijun Zhou, Qianjin Zhu, Stephanie Schorge, David C. Henshall

**Affiliations:** ^1^Department of Physiology and Medical Physics, RCSI University of Medicine and Health Sciences, Dublin, Ireland; ^2^FutureNeuro, the SFI Research Centre for Chronic and Rare Neurological Diseases, RCSI University of Medicine and Health Sciences, Dublin, Ireland; ^3^Department of Neuroscience, Physiology and Pharmacology, University College London, London, United Kingdom; ^4^Bicoll GmbH, Munich, Germany; ^5^Bicoll Biotechnology (Shanghai) Co., Ltd., Shanghai, China

**Keywords:** drug therapy, epilepsy, anti-seizure drugs, epileptiform activity, seizure

## Abstract

Drug-resistant epilepsy remains a significant clinical and societal burden, with one third of people with epilepsy continuing to experience seizures despite the availability of around 30 anti-seizure drugs (ASDs). Further, ASDs often have substantial adverse effects, including impacts on learning and memory. Therefore, it is important to develop new ASDs, which may be more potent or better tolerated. Here, we report the preliminary preclinical evaluation of BICS01, a synthetic product based on a natural compound, as a potential ASD. To model seizure-like activity *in vitro*, we prepared hippocampal slices from adult male Sprague Dawley rats, and elicited epileptiform bursting using high extracellular potassium. BICS01 (200 μM) rapidly and reversibly reduced the frequency of epileptiform bursting but did not change broad measures of network excitability or affect short-term synaptic facilitation. BICS01 was well tolerated following systemic injection at up to 1,000 mg/kg. However, we did not observe any protective effect of systemic BICS01 injection against acute seizures evoked by pentylenetetrazol. These results indicate that BICS01 is able to acutely reduce epileptiform activity in hippocampal networks. Further preclinical development studies to enhance pharmacokinetics and accumulation in the brain, as well as studies to understand the mechanism of action, are now required.

## Introduction

Epilepsy is one of the most common chronic neurological diseases, affecting up to 70 million people worldwide (1). Epilepsy is caused by complex biophysical, molecular, structural and functional changes to brain networks which lead to an imbalance between excitatory and inhibitory neurotransmission. This manifests clinically as spontaneous recurrent seizures (SRS) as well as co-morbidities which can include difficulties with learning, memory, sleep and mental health. Despite the availability of around 30 anti-seizure drugs (ASDs) (2), approximately 30% of people with epilepsy do not experience seizure freedom even with optimal treatment strategies (3). This figure may be as high as 3 out of 4 in patients with temporal lobe epilepsy (TLE) (4). In addition, many ASDs can have significant adverse effects which may severely impact a person's quality of life (5). These can include cognitive, sleep and mood impairments, which could also exacerbate the existing co-morbidities of epilepsy. It is therefore critical to develop novel therapeutic compounds for epilepsy which may be more effective in drug-resistant epilepsies and better tolerated by patients. Most ASDs are small molecules which typically act by interfering with neuronal ion channels and neurotransmitter systems to reduce excitability or boost inhibition in the brain (6). For example, ASDs can reduce voltage-gated sodium channel function (e.g., phenytoin, carbamazepine), increase voltage-gated potassium channel function (retigabine) or enhance GABA-mediated inhibition (e.g., benzodiazepines, tiagabine, vigabatrin). Other ASDs including levetiracetam, gabapentin and pregabalin act on pre-synaptic release machinery (6).

Here, we describe initial anti-seizure efficacy of BICS01. BICS01 is a novel synthetic putative anti-seizure compound, based on the structure of a natural product (7, 8). A therapeutic screen of the compound using the high potassium model of epileptiform activity in rat hippocampal slices (9, 10) showed that BICS01 mediated a powerful and reversible suppression of epileptiform activity. However, an initial *in vivo* test using the acute pentylenetetrazol (PTZ) seizure model showed no effect of BICS01 when administered systemically, 30 min prior to seizure induction. Taken together, BICS01 has promising anti-seizure effects with seemingly limited impact on normal hippocampal function, but further study is required to translate this finding to achieve *in vivo* efficacy and to ultimately build up a full preclinical profile for BICS01.

## Materials and Methods

### BICS01 Synthesis and Preparation

BICS01 was designed based on the structure of a natural product, and was purified by normal and reverse phase column chromatography with variable packing materials. It has a molecular weight of <250 DA. BICS01 was dissolved in 0.9% NaCl vehicle for experimental work.

### Ethics

*Ex vivo* experimental procedures in rats were carried out in accordance with the UK Animals (Scientific Procedures) Act 1986 and following the principles outlined in the ARRIVE (Animal Research Reporting *In vivo* Experiments) guidelines. *In vivo* procedures in mice were approved by the Research Ethics Committee of the Royal College of Surgeons in Ireland (REC-842), under license from the Ireland Health Products Regulatory Authority (AE19127/001).

### Brain Slice Preparation

Adult male Sprague-Dawley rats were anesthetized briefly with isoflurane and heavily with an overdose of sodium pentobarbital (IP), prior to cardiac perfusion with ice-cold oxygenated sucrose artificial cerebrospinal fluid (ACSF) slicing solution (in mmol/L: 205 sucrose, 10 glucose, 26 NaHCO_3_, 1.2 NaH_2_PO_4_.H_2_O, 2.5 KCl, 5 MgCl_2_, 0.1 CaCl_2_). 400 μm thick brain slices were prepared in the horizontal orientation with a Campden 7,000 smz vibratome (Campden Instruments, Loughborough, UK). Slices were submerged in recording ACSF (in mmol/L: 125 NaCl, 10 glucose, 26 NaHCO_3_, 1.25 NaH_2_PO_4_.H_2_O, 3 KCl, 2 CaCl_2_, 1 MgCl_2_) at room temperature and allowed to recover for at least 1 hour before recording.

### High Potassium Model of Epileptiform Activity

**Figure 1:**
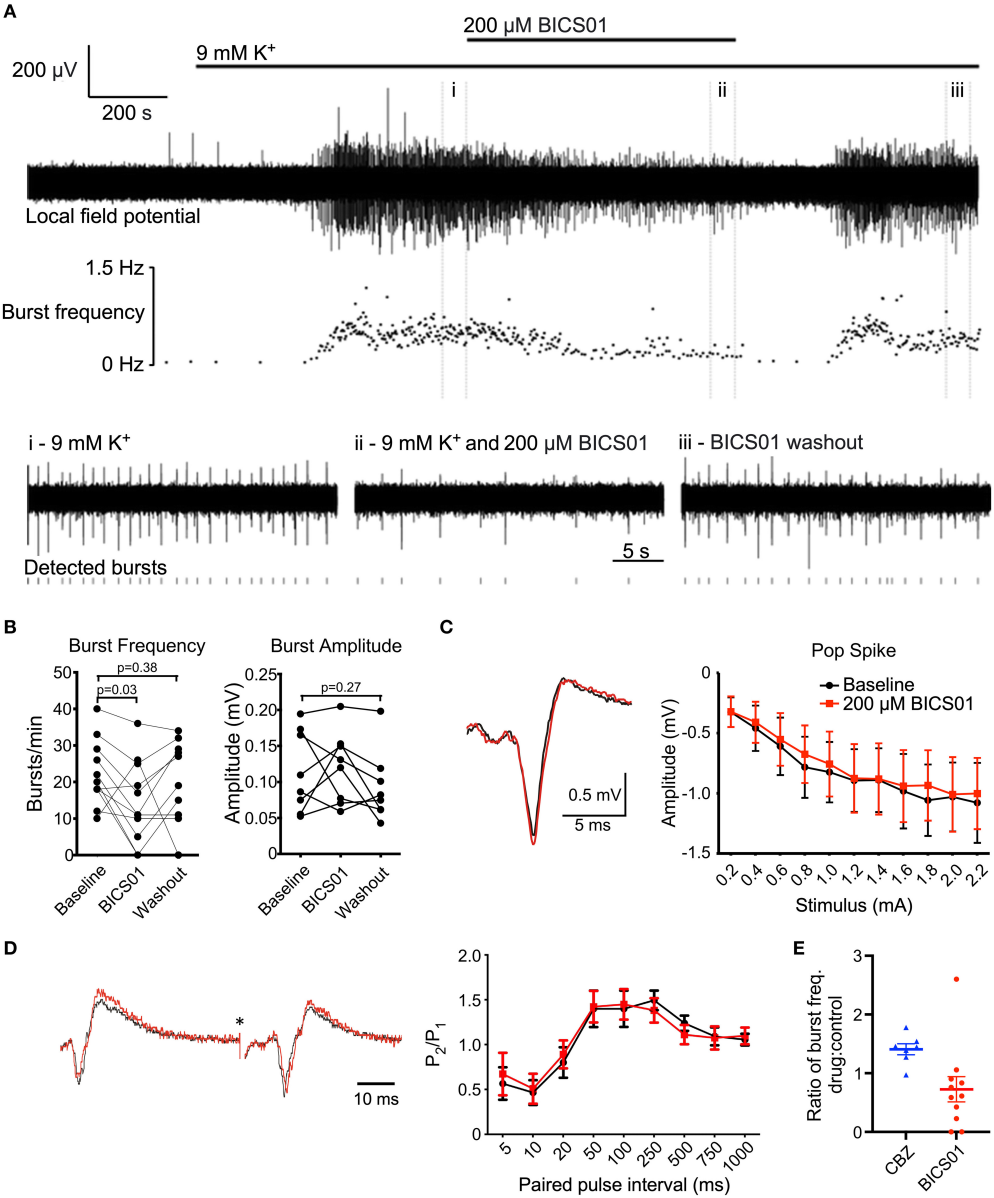
BICS01 reduces epileptiform bursting in hippocampal slices treated with 9 mM K^+^. **(A)** Representative trace showing epileptiform bursting in hippocampal CA1 elicited by 9 mM K^+^, before (i), during (ii) and after (iii) application of 200 μM BICS01. Raster plot shows instantaneous burst frequency throughout the experiment. **(B)** Summary data shows a significant decrease in burst frequency in the presence of 200 μM BICS01, which is reversed upon washout (RM one-way ANOVA with Dunnet's *post hoc* tests; p values marked on graph). Changes in burst amplitude caused by 200 μM BICS01 were not consistent between slices. **(C)** Stimulus-response curves and (D) paired-pulse facilitation are not altered by BICS01 (^*^truncated stimulus artifact, and amplitude of traces in **(D)** normalized to response to the first pulse). **(E)** 200 μM BICS01 mediates a stronger reduction in epileptiform bursting then 30 μM carbamazepine [CBZ data from (12)].

Slices were placed in a membrane chamber (10, 11) and perfused with oxygenated recording ACSF at 16 mL/min and ~34°C. Extracellular borosilicate glass micropipettes (~3 MΩ resistance) were filled with recording ACSF and placed into hippocampal CA1 and CA3 stratum pyramidale. Data were acquired using a MultiClamp 700B amplifier (Molecular Devices, CA, USA), National Instruments digitizer (BNC-2090A) and WinEDR software (John Dempster, Uni Strathclyde, UK). Baseline activity in ACSF + vehicle (0.9% NaCl) was recorded for at least 5 min, before epileptiform activity was induced by raising extracellular K^+^ to 9 mM (9). After the onset of epileptiform busting, the activity was allowed to stabilize for 10 min. The perfusate was then switched to normal ACSF with 9 mM K^+^ and 200 μM BICS01, and activity recorded for 10 min. The initial perfusate (ACSF with 9 mM K^+^ and vehicle) was used for washout. For identification of individual epileptiform bursts, raw LFP traces were low-pass filtered at 50 Hz to remove HFO components. Burst amplitude and frequency measurements were calculated from minute 9–10 in each condition (see also Figure 1). Burst amplitude was measured as the maximum peak-to-peak amplitude of an average waveform constructed from all individual bursts detected within this time window.

### Pentylenetetrazole (PTZ) Model of Acute Seizures

Adult male C57BL/6 mice were bred in house at the Biomedical Research Facility, RCSI, and originally sourced from Harlan, UK. Mice were treated with different doses (100, 300 and 1,000 mg/kg, IP) of BICS01, or 0.9% NaCl vehicle control. Thirty minutes after drug administration, mice received a convulsant dose of 80 mg/kg of PTZ (Sigma-Aldrich, Poole, UK) in 0.9% (w/v) NaCl via an i.p. injection to induce generalized seizures. Animals were placed individually in a clear chamber and video monitored for 30 min to record their behavior. After the observation period, animals were directly euthanized.

Videos of PTZ-induced seizures were analyzed offline using a modified Racine scale: 0, no change in behavior; 1, isolated myoclonic jerks; 2, atypical clonic seizure; 3, fully developed bilateral forelimb clonus; 4, tonic-clonic seizures with suppressed tonic phase with loss of righting reflex; 5, fully developed tonic-clonic seizure with loss of righting reflex (13). For each animal, the latency (in seconds) from the PTZ injection to the development of a first sign of seizure, clonic seizure and tonic-clonic seizure and the maximal seizure severity were recorded.

### Statistics

Averages are expressed as mean ± SEM. Data were tested for normality using Kolmogorov-Smirnov test. Data for Figure 1 were analyzed using repeated measures one-way ANOVA with Dunnett's multiple comparisons test. Statistical analysis was performed using GraphPad Prism (version 9; GraphPad, CA, USA).

## Results

### Physical Properties

BICS01 was purified (*via* ion exchange resin) to 95% purity, indicated by proton nuclear magnetic resonance (^1^H-NMR). The compound was dissolved in 0.9% NaCl aqueous solution, showing good thermodynamic stability (>5 mg/mL) and a measured kinetic solubility of 0.9 mg/mL. ^1^H-NMR monitoring studies indicated that BICS01 is highly stable at room temperature for at least 22 days, with no degradation during this time period. Physical properties of BICS01 are summarized in Table 1.

**Table 1:** Physical properties of BICS01.

**Acronym**	**Form**	**Purity**	**Solvent**	**Kinetic solubility**	**Thermodynamic solubility**	**pH**	**Stability after 22 d**
BICS01	Free form	95%	0.9% NaCl	1 mg/mL	>5 mg/mL	6.0	100%

### Effects of BICS01 in *Ex Vivo* Brain Slice Seizure Model

We first explored the effect of BICS01 in an acute brain slice model of epileptiform activity. Seizure-like activity was induced in hippocampal slices from rats by the application of 9 mM extracellular K^+^ (Figure 1A). This reliably induced epileptiform bursting in the hippocampus (22.3 ± 2.7 bursts/min; total of 9 slices across 6 rats) in the presence of the 0.9% NaCl vehicle.

Addition of 200 μM BICS01 for 10 min reduced burst frequency by ~35% to 14.6 ± 3.4 bursts/min (Figures 1A,B; repeated measures (RM) one-way ANOVA with Dunnet's *post hoc* test, *P* = 0.03). This effect was reversed after 10 min of washout, with burst frequency restored to a level comparable to baseline (Dunnet's *post hoc* test, *P* = 0.38). This suggests a reversible suppression of epileptiform activity in the presence of BICS01. Changes to burst amplitude in the presence of BICS01 were inconsistent between slices (Figure 1B).

To begin to probe the mechanism(s) underlying this anti-seizure effect, we stimulated the Schaffer collateral pathway of hippocampal slices and recorded population local field potentials in CA1 stratum pyramidale and stratum radiatum. We measured both stimulus-response curves and paired-pulse facilitation in baseline conditions (3 mM extracellular K^+^). The addition of 200 μM BICS01 had no effect on either paradigm (Figures 1C,D), indicating that its mechanism of action is unlikely due to a direct effect on hippocampal network excitability or short-term plasticity.

### Effect of BICS01 on PTZ-Induced Seizures in Mice

**Figure 2:**
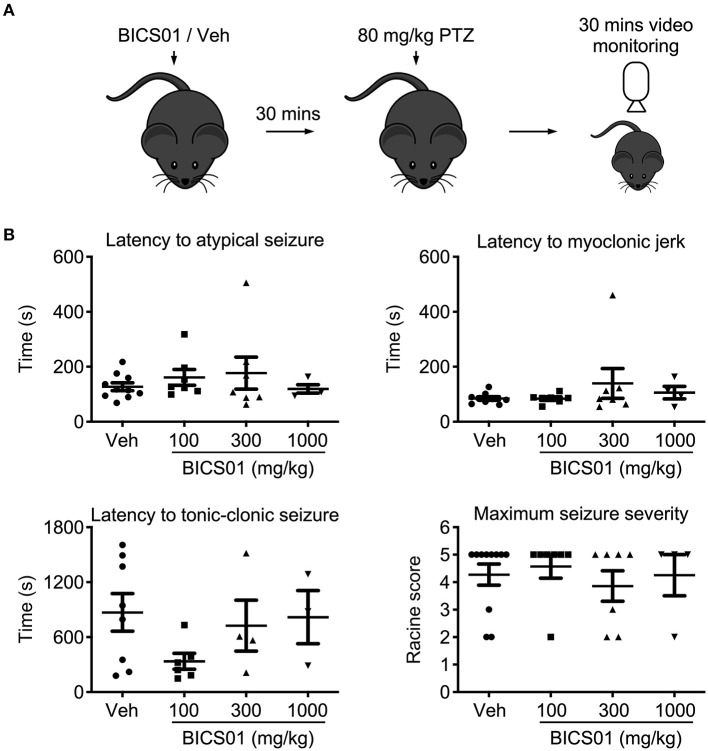
Acute systemic application of BICS01 has limited effects in an acute *in vivo* seizure model. **(A)** Experimental overview. BICS01 (100, 300, or 1,000 mg/kg) or 0.9% NaCl vehicle were administered via IP injection 30 min prior to acute seizure induction with pentylenetetrazol. Mice were video monitored for the following 30 min to observe the behavioral manifestation of acute seizures. **(B)** Latencies to atypical seizure, myoclonic jerk and tonic-clonic seizure, as well as maximum seizure severity reached, were all unchanged.

Having demonstrated efficacy of BICS01 in *ex vivo* brain slices, we then performed an initial dose-range finding study *in vivo*, using the systemic pentylenetetrazol (PTZ) model of acute seizures in mice. This is a standard test used as an initial screen for anticonvulsant effects of putative ASDs (14) BICS01 was injected systemically 30 min prior to seizure induction with PTZ. Mice were observed during this time and showed no signs of toxic or adverse reaction. 80 mg/kg (IP) PTZ reliably induced seizures in mice, but BICS01 did not alter the onset of myoclonic jerks, atypical or tonic-clonic seizures relative to vehicle control, at all doses tested (100, 300, 1,000 mg/kg; Figure 2). Maximal seizure severity was also unaffected. This suggests that despite promising anti-epileptiform activity effects in *ex vivo* brain slices, BICS01 might be not optimal to increase brain uptake and ultimately to translate the compound for clinical use.

## Discussion

Drug-resistant epilepsy is a significant healthcare and socioeconomic burden (4, 15), and there is an urgent need to discover new compounds with anti-seizure properties. BICS01 represents a novel synthetic compound, which mediated powerful and reversible anti-seizure effects using an *ex vivo* hippocampal slice seizure model. However, limited efficacy in the PTZ model suggests that further study is required to establish or optimize the anti-seizure effects of BICS01 *in vivo* following acute systemic application.

BICS01 is based upon a natural product and was synthesized in gram scale. Purity and compound integrity were measured with 1H NMR, 13C NMR and LC-MS. The most important finding in the present study was that BICS01 displayed rapid and reversible anti-seizure effects in an *ex vivo* slice model of epilepsy. First, BICS01 significantly reduced both the frequency and amplitude of epileptiform bursts in the model. Indeed, bursts were almost completely absent within 100 min of drug application to the slice. This effect is predicted to be stronger than that of carbamazepine (CBZ; Figure 1E) which we observed previously in a similar model (12). Since CBZ remains a frontline ASD in current clinical use, this raises the prospect that BICS01 might be able to perform as well as, or better than, current anti-seizure drugs. It should however be noted that the concentration of BICS01 used (200 μM) is greater than that of CBZ (30 μM). Further dose-ranging studies will be required to ascertain the minimal concentration of BICS01 to mediate anti-seizure effects in our brain slice model.

The present study also began to explore the mechanism underlying the observed anti-seizure effect. Using *ex vivo* brain slices in normal (non-epileptiform) conditions, we observed no effect of BICS01 on either baseline hippocampal excitability or paired-pulse facilitation. These data suggest that physiological brain circuit function may be spared by BICS01. Similarly, preliminary patch clamp data showed no effect on pyramidal neuron firing during the slice seizure models, suggesting that BICS01 spares normal pyramidal neuron function (data not shown). As adverse effects on learning and memory are amongst the most common detrimental effects of current ASDs (5), this preserved cellular and network hippocampal function may represent a substantial advantage of BICS01. *In vivo* behavioral testing to assess learning and memory will be a critical aspect of the later pre-clinical safety testing.

We saw limited efficacy of BICS01 on seizures induced acutely *in vivo* using PTZ. This most likely reflects it being a highly polar molecule that may not readily cross the blood-brain barrier (BBB) within the set time limit (30 min before PTZ application), which regulates the passage of systemically delivered macromolecules into the brain (16). Optimal compounds will show improved pharmacokinetics. Such bioavailability concerns may, however, be less important in chronic epilepsy, where the BBB is more permeable (17, 18). In this case, spontaneous recurrent seizures (SRS) can cause a temporary increase in BBB permeability, allowing transient passage of certain molecules into the brain. It may also be that BICS01 is only efficacious in particular seizure models and highlights the value of different models which capture diverse disease etiologies. The high potassium slice model used here elicits seizure-like activity through a global depolarisation of neurons (9), whereas our *in vivo* approach used PTZ, a non-competitive antagonist of the GABA-A receptor (19). Notably, rodent models do not always show strong predictive validity (20, 21) (i.e., - ASDs in common clinical use are ineffective in one or more *in vivo* seizure screen). Further, it should be noted that seizures induced acutely with PTZ, whilst advantageous in terms of throughput, do not model the spontaneous recurrent seizures seen in drug-resistant epilepsy (14). Further study will be required to determine whether BICS01 has model-specific actions, though it is likely that this would be performed with optimal compounds which are designed to better and more quickly cross the BBB. Finally, for our screen BICS01 was only administered in one dose, 30 min prior to seizure induction with PTZ. It could be that longer-term dosing is required to build up sufficient concentration of BICS01 in the brain to mediate its anti-seizure effect *in vivo*.

### Future Studies

Whilst our data provide compelling evidence to support the preclinical development of BICS01, further study is required to interrogate its mechanism of action and efficacy in other models. A key question is whether BICS01 can readily cross the BBB. Our *in vivo* data, coupled with the ready solubility of the molecule in saline, suggest that this may not be the case. Future inventions will target these critical parameters in order to eventually seek full regulatory approval as potential novel treatment options. During the development of these compounds, it will be critical to test these, and BICS01, in different models both *in vitro* and *in vivo*. BICS01 is likely to have different efficacy in models with different mechanisms and perhaps with milder seizure phenotypes. These studies will allow us to better understand possible mechanisms and clinical indications.

### Summary

BICS01 is a highly promising novel anti-seizure compound, with strong efficacy in a brain slice model and no apparent impact on normal hippocampal function. This provides a clear basis to progress the development of optimal compounds for full preclinical study and *in vivo* applications based on the current disclosure.

## Data Availability Statement

The original contributions presented in the study are included in the article/supplementary material, further inquiries can be directed to the corresponding author.

## Ethics Statement

The animal study was reviewed and approved by Research Ethics Committee of the Royal College of Surgeons in Ireland.

## Author Contributions

GM, MH, and TH generated experimental data. KL, HG, YZ, and QZ developed the compound. GM, KL, SS, and DH conceived the study. GM wrote the manuscript. All authors approved the final manuscript.

## Funding

This publication has emanated from research conducted with the financial support of the European Union's ‘Seventh Framework’ Programme (FP7) under Grant Agreement Number 602130. Additionally, this publication has emanated from research supported in part by a research grant from Science Foundation Ireland (SFI) under Grant Number 16/RC/3948 and co-funded under the European Regional Development Fund and by FutureNeuro industry partners. GM was supported by a Marie Skłodowska-Curie Actions Individual Fellowship (‘EpimiRTherapy’, H2020-MSCA-IF-2018 840262) and an Emerging Leader Fellowship Award from Epilepsy Research UK (grant reference F2102 Morris). KL was grateful for partial public funding by the German Ministry of Education and Research (BMBF 13GW0048).

## Conflict of Interest

BICS01 was developed by Bicoll GmbH. KL is a full-time employee of Bicoll GmbH and HG, YZ, and QZ are full-time employees of Bicoll Biotechnology (Shanghai) Co., Ltd. The remaining authors declare that the research was conducted in the absence of any commercial or financial relationships that could be construed as a potential conflict of interest.

## Publisher's Note

All claims expressed in this article are solely those of the authors and do not necessarily represent those of their affiliated organizations, or those of the publisher, the editors and the reviewers. Any product that may be evaluated in this article, or claim that may be made by its manufacturer, is not guaranteed or endorsed by the publisher.
